# Early versus delayed DOAC after ischaemic stroke in atrial fibrillation: 1-year outcomes in the TIMING study and in concurrent practice

**DOI:** 10.1093/esj/aakag010

**Published:** 2026-02-27

**Authors:** Oskar Fasth, Jonas Oldgren, Tatevik Ghukasyan Lakic, Ziad Hijazi, Bo Norrving, Per Wester, Signild Åsberg

**Affiliations:** Department of Medical Sciences, Uppsala University, Uppsala, Sweden; Department of Medical Sciences, Uppsala University, Uppsala, Sweden; Uppsala Clinical Research Centre, Uppsala University, Uppsala, Sweden; Uppsala Clinical Research Centre, Uppsala University, Uppsala, Sweden; Department of Medical Sciences, Uppsala University, Uppsala, Sweden; Department of Clinical Sciences, Lund University, Lund, Sweden; Department of Public Health and Clinical Science, Umeå University, Umeå, Sweden; Department of Clinical Sciences, Danderyd Hospital, Karolinska Institutet, Stockholm, Sweden; Department of Medical Sciences, Uppsala University, Uppsala, Sweden

**Keywords:** anticoagulants, antithrombins, apixaban, atrial fibrillation, dabigatran, edoxaban, embolic stroke, ischaemic stroke, rivaroxaban

## Abstract

**Introduction:**

A recent meta-analysis, including the TIMING study, found favourable short-term outcomes after early initiation of a direct-acting oral anticoagulant (DOAC), as compared to delayed initiation, following acute ischaemic stroke in patients with atrial fibrillation. We aimed to investigate 1-year outcomes after early vs delayed DOAC initiation in the TIMING study population and a concurrent observational cohort.

**Patients and methods:**

In TIMING, adults with atrial fibrillation were randomised to initiation of DOAC either within 4 days or at 5–10 days after acute ischaemic stroke. The non-randomised cohort comprised patients with acute ischaemic stroke and atrial fibrillation, observed in the Swedish Stroke Register concurrently with the TIMING study and initiating DOAC within 4 or 5–10 days, respectively. Outcomes in both populations were acute ischaemic stroke, symptomatic intracerebral haemorrhage or death, as a composite and individually, analysed using Cox regression models, with adjustment for risk factors in the observational cohort.

**Results:**

In the TIMING population (*n* = 888), the composite outcome at 365 days occurred in 64/450 patients (14.2%) with early DOAC initiation and 66/438 (15.1%) with delayed, unadjusted hazard ratio 0.92 (95% CI, 0.66–1.30). In the observational cohort (*n* = 8951), the composite outcome at 365 days occurred in 1227/6671 (18.4%) patients initiating DOAC early and 511/2280 (22.4%) in the delayed group, adjusted hazard ratio 0.80 (0.69–0.91).

**Discussion:**

At 1-year follow-up, early initiation of DOAC remained safe and effective with no increase in symptomatic intracerebral haemorrhage. The results support early DOAC initiation in patients with acute ischaemic stroke and atrial fibrillation.

## Graphical Abstract

**Graphical Abstract:**
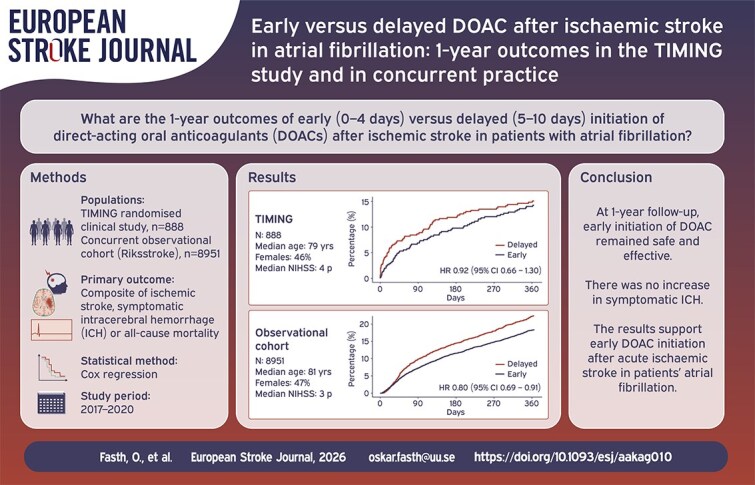

## Introduction

Atrial fibrillation is a common cause of ischaemic stroke, and preventive treatment with anticoagulants is recommended for most patients with atrial fibrillation in current guidelines.^1,2^ The optimal time window to start anticoagulants following an ischaemic stroke has been a matter of debate.^3,4^ Four randomised trials, Timing of Oral Anticoagulant Therapy in Acute Ischaemic Stroke with Atrial Fibrillation (TIMING), Early vs Later Anticoagulation for Stroke with Atrial Fibrillation (ELAN), Optimal Timing of Anticoagulation after Acute Ischaemic Stroke with Atrial Fibrillation (OPTIMAS) and Optimal Delay Time to Initiate Anticoagulation after Ischaemic Stroke in Atrial Fibrillation (START) have recently investigated early vs delayed initiation of direct-acting vitamin K oral anticoagulants (DOAC) on composite outcomes of adverse events after acute ischaemic stroke with atrial fibrillation.^5–8^ A recent meta-analysis of these 4 trials^9^ found early initiation of DOAC treatment superior to delayed initiation for the prevention of recurrent ischaemic stroke at 30 days after index stroke, without evidence of increased risk of symptomatic intracranial or extracranial haemorrhage. Of note, at 90 days after index stroke there was no significant benefit of early initiation of DOAC.

The long-term implications of early initiation of DOAC in the acute phase have not been studied so far.^9^ It may be hypothesised that differences in outcomes, particularly the risk of death, could arise beyond the first months, given that an association between severity of post-stroke disability and, eg, long-term risk of death is well-known.^10,11^

Evidence from randomised controlled trials is the gold standard when evaluating medical interventions. Observational studies can provide additional assurance of external validity when analysed alongside data from randomised trials.^12,13^

### Aims

Our main aim was to investigate the 1-year outcomes of early vs delayed DOAC initiation in the TIMING study population. Additionally, we investigated the same outcomes in an observational cohort of patients registered in the Swedish Stroke Register (Riksstroke), allowing for comparison between the randomised study participants and routine clinical practice.

## Methods

### Study design

This study was a pre-specified secondary analysis of data from the randomised TIMING study, with the addition of a companion national, registry-based, observational cohort study for comparative purposes.

### Data sources

#### The Swedish Stroke Register (Riksstroke)

Riksstroke is a long-running healthcare quality register, covering all 72 stroke units and approximately 95% of all adult stroke cases in Sweden. Its main purpose is to collect data for quality assurance and to be able to compare healthcare interventions for stroke over time and between hospitals. Patients are informed of the data collection and assent on an opt-out basis. The data can retrospectively be made available for research purposes with applicable ethical permission.^5,14^

#### The National Cause of Death Register

This mandatory national health data register, hosted by the Swedish National Board of Health and Welfare, collects data on cause of death for all persons who die in Sweden.^15^

#### The TIMING study

The protocol of the TIMING study (NCT02961348) has been described in detail elsewhere.^16^ In brief, TIMING was a pragmatic, open-label, non-inferiority study, utilising the Riksstroke infrastructure to collect follow-up data at 90 days. It was a multi-centre study, conducted at 34 Swedish stroke units. The decision to screen patients for participation was made by the local primary investigator or delegated sub-investigators. Patients aged 18 years and older, with atrial fibrillation and recent stroke (<72 h from symptom onset), were randomised to either early (≤4 days) or delayed (5–10 days) initiation of DOAC treatment, between 2 April 2017 and 30 December 2020.

### Populations

#### TIMING population

All patients in the TIMING study intention-to-treat population (*n* = 888) were included in the present study, and are henceforth referred to as the “TIMING population.”

#### Observational cohort

For the observational cohort, we retrospectively collected data on patients registered in Riksstroke,^14^ hospitalised with acute ischaemic stroke during the TIMING study period (1 January 2017–31 December 2020), who did not participate in the TIMING study. As in the TIMING study, these patients were aged 18 years or older and had a diagnosis of atrial fibrillation made before or during the index hospitalisation. For the observational study, patients who died during the index hospitalisation were excluded, as data on pharmaceutical treatment are not collected for deceased patients in Riksstroke. Patients without a valid Swedish personal identification number were likewise excluded, as linkage with the national registries would have been impossible. To align with the TIMING study population, we only included patients where DOAC was initiated within 10 days after symptom onset of the index stroke, and retrospectively divided the observational cohort into 2 treatment groups according to early (0–4 days) vs delayed (5–10 days) initiation of DOAC. At the time of treatment, neither patients nor treating physicians were aware that the data collected were going to be used for research purposes. Treatment decisions were made at the discretion of the treating physician, reflecting routine clinical practice.

### Baseline data

Baseline data for both populations were collected from Riksstroke.

### Outcomes

Study outcomes in both populations were the composite of recurrent ischaemic stroke, symptomatic intracerebral haemorrhage or all-cause mortality and the individual components of this composite.

#### TIMING population

For the TIMING population, all events were recorded starting from randomisation. For each outcome, patients were followed until an event occurred or 365 days had passed.

Data on outcome events, including all-cause mortality, for the first 28 days after randomisation were collected from a specific TIMING study case report form within the Riksstroke framework. Between days 29 and 365, data on recurrent ischaemic stroke and symptomatic intracerebral haemorrhage were retrieved from routinely collected data in Riksstroke. Data on all-cause mortality between days 29 and 90 were also retrieved from Riksstroke, and from day 91 until 365 days from the National Cause of Death Register.^15^ All events until day 90 were adjudicated by telephone interviews and/or medical chart reviews.

#### Observational cohort

For the observational cohort, data regarding events were possible to retrieve only after discharge from the index hospitalisation, but to harmonise with analyses in the TIMING population, calculation of time-to-event started from the index event. For each outcome, patients were followed until an event occurred or 365 days had passed.

For the entire follow-up period, ie, from discharge from the index hospitalisation until 365 days, data on recurrent ischaemic stroke and symptomatic intracerebral haemorrhage were collected from Riksstroke, while data on all-cause mortality were collected from the National Cause of Death Register.

### Statistical analysis

Baseline data for both study populations were analysed descriptively, with numeric variables summarised using median and interquartile range, and categorical variables summarised using counts and percentages. In the present publication, event rates are described as numbers and proportions of patients with events, and illustrated using Kaplan–Meier plots. Levels of missing data are reported for variables where it exceeded > 2%.

Statistical analyses for the composite outcome, as well as for its separate components, were performed using proportional-hazards Cox regression analysing time to first event. All statistical analyses were performed using R version 4.4.1.

#### Models

For the randomised TIMING population, the models included treatment group as the only covariate.

For the observational cohort, 2 models were fitted per outcome, 1 crude and 1 adjusted for age, sex, stroke severity (defined as National Institutes of Health Stroke Scale score at admission), reperfusion therapy (yes/no), prior antithrombotics treatment, antihypertensives, prior atrial fibrillation, prior stroke/transient ischaemic attack and diabetes.

#### Ethical approval

The TIMING study was approved by the Regional Ethics Committee in Uppsala, Sweden (reference number 2016/359). The companion observational study was approved by the Swedish Ethical Review Authority (reference number 2021-00430).

## Results

### TIMING population

**Figure 1:**
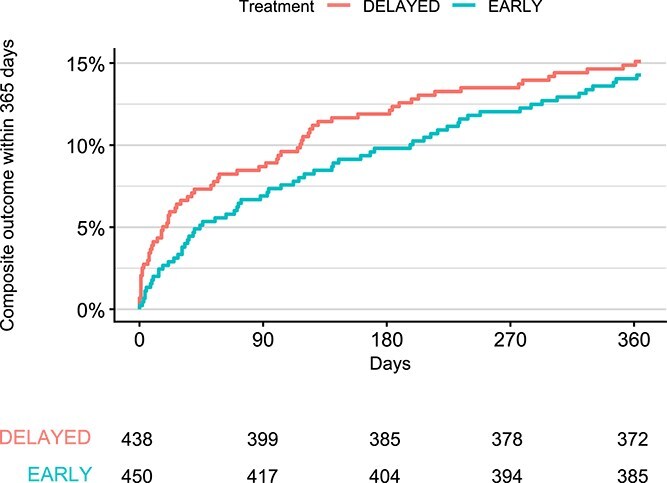
Kaplan–Meier plot of the composite outcome event at 365 days, TIMING population.

The main results from the randomised TIMING study are previously published.^5^ The population consists of 888 patients, with 450 randomised to the early initiation group, and 438 to the delayed group. In this pre-specified secondary analysis of the TIMING study, the composite outcome of death, recurrent ischaemic stroke or symptomatic intracerebral haemorrhage until 365 days occurred in 64/450 (14.2%) individuals in the early group and 66/438 (15.1%) in the delayed group (Figure 1), with a hazard ratio of 0.92 (95% CI, 0.66–1.30). Death occurred in 44/450 (9.8%) of patients in the early group, and 50/438 (11.4%) in the delayed group (Figure S2), with a HR for all-cause mortality of 0.84 (95% CI, 0.56–1.26). A first recurrence of ischaemic stroke occurred in 25/450 (5.6%) of patients in the early group and 28/438 (6.4%) in the late group (Figure S4), with an HR of 0.85 (95% CI, 0.50–1.46). There were a total of 2/450 (0.4%) symptomatic intracerebral haemorrhages in the early group, both occurring between days 91 and 365. No symptomatic intracerebral haemorrhages were observed in the delayed group (*n* = 438) during the entirety of the follow-up period.

### Observational cohort

During 2017–2020, 19,096 patients were registered in the Riksstroke register with an incident stroke and a diagnosis of atrial fibrillation, made prior or during the initial hospitalisation. After removal of patients who did not fulfil eligibility criteria and after cross-linkage with the national registries, the final observational cohort comprised 8951 patients (Figure S1), of which 6671 had initiated DOAC early (0–4 days from symptom onset of the index stroke) and 2280 late (5–10 days from symptom onset of the index stroke). While baseline characteristics in the observational cohort were generally similar between the early and delayed groups, fewer patients in the delayed group had a previously known diagnosis of atrial fibrillation and/or previous treatment with anticoagulants, and more patients in the delayed group had received reperfusion therapy. The delayed group had more days in hospital during the first 90 days, mainly driven by a longer index hospitalisation stay (Table 1).

**Table 1:** Baseline characteristics, observational cohort, with the characteristics of the TIMING study population for comparison. Missing data are reported for variables where it exceeds 2%.

	**Observational cohort**			**TIMING study**
	**Early**	**Delayed**	**All**	**All**
	*n* = 6671	*n* = 2280	*n* = 8951	*n* = 888
**Age, median (IQR)**	81 (74–86)	81 (74–87)	81 (74–86)	78.9 (72.8–85.4)
***Age—subgroups, n (%)***				
** 18–64**	424 (6.4%)	156 (6.8%)	580 (6.5%)	88 (9.9%)
** 65–74**	1318 (19.8%)	422 (18.5%)	1740 (19.4%)	200 (22.5%)
** 75–84**	2806 (42.1%)	928 (40.7%)	3734 (41.7%)	368 (41.4%)
** 85+**	2123 (31.8%)	774 (33.9%)	2897 (32.4%)	232 (26.1%)
**Female sex, *n* (%)**	3033 (45.5%)	1142 (50.1%)	4175 (46.6%)	410 (46.2%)
**Previously known atrial fibrillation, *n* (%)**	4727 (70.9%)	1183 (51.9%)	5910 (66.0%)	436 (49.1%)
**Prior stroke, *n* (%)**	1445 (21.7%)	428 (18.8%)	1873 (20.9%)	155 (17.5%)
**Prior TIA, *n* (%)**	740 (11.1%)	190 (8.3%)	930 (10.4%)	62 (7%)
**Diabetes, *n* (%)**	1655 (24.8%)	524 (23%)	2179 (24.3%)	172 (19.4%)
**Active smoking, *n* (%)**	503 (7.5%)	183 (8%)	686 (7.7%)	71 (8%)
**-- Missing, *n* (%)**	913 (13.7%)	368 (16.1%)	1281 (14.3%)	
**ADL-independent before stroke, *n* (%)**	5722 (85.8%)	1944 (85.3%)	7666 (85.6%)	819 (92.2%)
**-- Missing, *n* (%)**	183 (2.7%)	91 (4%)	274 (3.1%)	
***Living conditions before stroke***				
** Nursing home, *n* (%)**	477 (7.2%)	167 (7.3%)	644 (7.2%)	44 (5%)
** Other, *n* (%)**	13 (0.19%)	4 (0.18%)	17 (0.19%)	0 (0%)
** Own home w assistance, *n* (%)**	1352 (20.3%)	436 (19.1%)	1788 (20%)	113 (12.7%)
** Own home w/o assistance, *n* (%)**	4814 (72.2%)	1665 (73%)	6479 (72.4%)	730 (82.2%)
***Pharmaceutical treatment on admission***				
**Antihypertensives, *n* (%)**	5455 (81.8%)	1766 (77.5%)	7221 (80.7%)	671 (75.6%)
**Statins, *n* (%)**	2588 (38.8%)	759 (33.3%)	3347 (37.4%)	327 (36.8%)
**Antiplatelet (without additional oral anticoagulant), *n* (%)**	963 (14.4%)	534 (23.4%)	1497 (16.7%)	193 (21.7%)
**Dual antiplatelet treatment, *n* (%)**	38 (0.57%)	30 (1.3%)	68 (0.76%)	6 (0.68%)
**Single antiplatelet treatment, *n* (%)**	1103 (16.5%)	552 (24.2%)	1655 (18.5%)	193 (21.7%)
**Warfarin, *n* (%)**	472 (7.1%)	246 (10.8%)	718 (8%)	65 (7.3%)
**Warfarin initial INR, median (IQR)**	2.1 (1.6–2.5)	2.3 (1.7–2.8)	2.2 (1.6–2.7)	1.9 (1.5–2.4)
**-- Warfarin-treated, missing INR, *n* (%)**	305 (64.6%)	131 (53.3%)	436 (60.7%)	0 (0%)
**(Any) DOAC, *n* (%)**	3276 (49.1%)	413 (18.1%)	3689 (41.2%)	175 (19.7%)
**Apixaban, *n* (%)**	2379 (35.7%)	273 (12%)	2652 (29.6%)	111 (12.5%)
**Dabigatran, *n* (%)**	293 (4.4%)	44 (1.9%)	337 (3.8%)	26 (2.9%)
**Edoxaban, *n* (%)**	52 (0.78%)	16 (0.7%)	68 (0.76%)	2 (0.23%)
**Rivaroxaban, *n* (%)**	552 (8.3%)	80 (3.5%)	632 (7.1%)	36 (4.1%)
**No antithrombotic treatment**	1931 (28.9%)	1056 (46.3%)	2987 (33.4%)	455 (51.2%)
**NIHSS on admission, median (IQR)**	3 (1–6)	5 (2–11)	3 (1–8)	4 (2–9)
***NIHSS on admission—subgroups, n (%)***				
** 0–3**	2383 (35.7%)	589 (25.8%)	2972 (33.2%)	351 (39.5%)
** 4–5**	635 (9.5%)	175 (7.7%)	810 (9%)	126 (14.2%)
** 6–10**	737 (11%)	317 (13.9%)	1054 (11.8%)	157 (17.7%)
** 11–15**	305 (4.6%)	215 (9.4%)	520 (5.8%)	80 (9%)
** 16+**	249 (3.7%)	208 (9.1%)	457 (5.1%)	79 (8.9%)
**Missing NIHSS, *n* (%)**	2362 (35.4%)	776 (34%)	3138 (35.1%)	95 (10.7%)
**Thrombectomy, *n* (%)**	253 (3.8%)	228 (10%)	481 (5.4%)	121 (13.6%)
**Thrombolysis, *n* (%)**	652 (9.8%)	347 (15.2%)	999 (11.2%)	252 (28.4%)
**No reperfusion therapy, *n* (%)**	5849 (87.7%)	1808 (79.3%)	7657 (85.5%)	575 (64.8%)
**Length of hospital stay during index hospitalisation, median (IQR)**	7 (4–13)	11 (8–19)	8 (4–15)	6 (3–13)
**Length of hospital stay within 90 days, median (IQR)**	8 (4–16)	13 (8–23)	10 (5–18)	7 (3–15)

**Figure 2:**
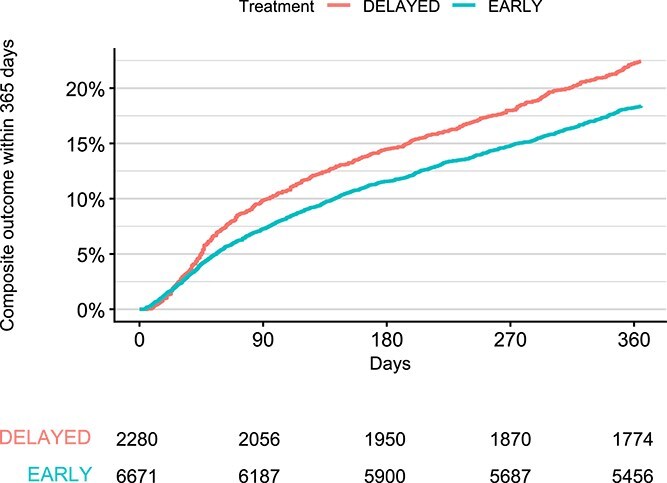
Kaplan–Meier plot of the composite outcome event at 365 days, observational cohort.

A total of 1738/8951 (19.4%) patients in the observational cohort experienced a first occurrence of the composite outcome of death, recurrent ischaemic stroke or symptomatic intracerebral haemorrhage until 365 days (Figure 2), with 1227/6671 (18.4%) in the early group and 511/2280 (22.4%) in the delayed group. The unadjusted HR for the composite outcome was 0.80 (95% CI, 0.72–0.89) and adjusted HR was 0.80 (95% CI, 0.69–0.91) for early vs delayed DOAC start. In the early group, 1004/6671 (15.1%) of patients died during follow-up, and 451/2280 (19.8%) died in the delayed group (Figure S3), with an unadjusted HR of 0.74 (95% CI, 0.66–0.82) and adjusted 0.75 (95% CI, 0.64–0.87). Recurrent ischaemic stroke occurred in 291/6671 (4.4%) and 81/2280 (3.6%) in the early and delayed groups, respectively (Figure S5), yielding an unadjusted HR of 1.20 (95% CI, 0.93–1.53) and adjusted 1.02 (95% CI, 0.75–1.39). Symptomatic intracerebral haemorrhage occurred in 33/6671 (0.49%) patients in the early group, and 10/2280 (0.44%) in the delayed group (Figure S6), with an unadjusted HR of 1.09 (95% CI, 0.54–2.21) and adjusted 0.84 (95% CI, 0.36–1.93).

Short-term outcome events in the observational cohort (until 90 days) are described in Table S1.

## Discussion

In this 1-year follow-up of patients in both the randomised TIMING study and the concurrent observational cohort, early initiation of DOAC treatment was as safe and effective as delayed initiation in patients with acute ischaemic stroke and atrial fibrillation. The results of the pre-specified extended follow-up of the TIMING study are in line with the previously reported primary outcome at 90 days, where early initiation was shown non-inferior to delayed start of DOAC.^5^

In the observational cohort, the results favoured early initiation of DOAC after ischaemic stroke, with significantly lower rates of the composite outcome of death, recurrent ischaemic stroke or symptomatic intracerebral haemorrhage at 1-year follow-up.

The findings of this study add further weight to the growing body of evidence that early initiation of DOACs after stroke in patients with atrial fibrillation is at least as safe and effective as delaying initiation, something that until very recently was controversial, giving rise to marked differences across guidelines and regional practice.^4^ Early initiation offers several practical advantages. Integrating DOAC initiation into the initial intervention package provided to acute stroke patients during their hospital stay may facilitate shorter hospitalisations, reduce time with no antithrombotic treatment or with less effective antiplatelet agents, as well as mitigate the need for early outpatient visits. In addition to potential healthcare cost savings, this approach decreases information complexity for patients, caregivers and next-of-kin, it streamlines the discharge process and increases the likelihood that the treatment will be both offered and appropriately understood and adhered to by patients.

Concerns of an increased risk of haemorrhagic transformation, historically observed with older classes of anticoagulants^17,18^ and often cited as the primary rationale for delaying DOAC initiation in ischaemic stroke,^4,19^ are not supported by findings in the present study, nor in the recently published CATALYST meta-analysis.^9^ Consequently, for the vast majority of patients with stroke and atrial fibrillation, these concerns should not influence the timing of DOAC initiation.

Not only were baseline characteristics similar between the TIMING population and the observational cohort but outcome event rates were also similar, adding strong support to the generalisability of the randomised study results. Among differences between the populations, it is worth noting the shorter length of index event hospital stay in the TIMING population, which may be related to having a set protocol for DOAC initiation for patients participating in the randomised study. The proportion of patients that had undergone reperfusion therapy also differed, with more patients in the randomised study having received either thrombolysis or thrombectomy. Patients in the TIMING study were disproportionally recruited at comprehensive stroke centres, which may have higher rates of reperfusion treatment. Another possible reason is that they were more likely to be treated at a dedicated stroke unit, increasing the chance that they would be screened for study enrolment. Being monitored at a dedicated stroke unit might also increase the likelihood that a previously undiagnosed atrial fibrillation would be detected, which might contribute to enrolment, as well as explain the difference in previously known atrial fibrillation seen between the populations.

A main strength of this analysis was the combination of a randomised study population with a large, geographically and temporally concurrent observational cohort. Another strength was the use of a disease-specific national quality registry, Riksstroke, for collection of baseline characteristics, and the use of mandatory public registers with complete coverage of all hospitalisations in Sweden for follow-up measures. This not only facilitated the conduct of the randomised clinical study by limiting the need for study-specific procedures, it also simplified long-term follow-up and enabled outcome comparisons between the randomised study population and the observational cohort using the same data sources and variables, strengthening external generalisability.

The registers used in this study, Riksstroke and the National Cause of Death Register, are both of high quality and the number of patients lost to follow-up was likely very low. Registration in the National Cause of Death Registry is mandatory for all deaths occurring in Sweden, with coverage errors estimated to be very low.^20^ For Riksstroke, coverage is approximately 95% when compared to a first diagnosis of stroke in the National Patient Register.^14^

This study has limitations; for one, the TIMING study recruited fewer patients than initially planned and as a consequence may not have sufficient power to detect small differences between groups. In the original 90-day follow-up, there were no symptomatic intracerebral bleeding events at all,^5^ an outcome that in previous studies is an important driver of difference between early and delayed initiation of anticoagulants.^4^ Of note, in the 3 other randomised clinical trials, ELAN, OPTIMAS and START, cases of symptomatic intracerebral bleeding were likewise very few, supporting the safety of early initiation of DOAC treatment.^6–9^

Almost all variables in Riksstroke have some degree of missing data, but for all except 3 of the variables (NIHSS at admission, smoking status and independence at admission), levels of missing data were below 2%, which was deemed inconsequential. Of the 3 variables with higher levels of missing data, the NIHSS score at admission was notable, with 10.7% missing in the TIMING population and 35.1% in the observational cohort, but with similar numbers between the treatment arms within each of the populations. This is a possible source of bias, mainly for the observational cohort, as stroke severity was likely a factor in the decision when to initiate treatment in the non-randomised population.

Although we aimed to align the observational cohort closely with the TIMING study regarding eligibility criteria and outcome measures, a full target trial emulation was not feasible. A key limitation was the inability—due to the Riksstroke design—to capture recurrent cerebrovascular events occurring during the index hospitalisation in the observational cohort. It is likely that some events did occur within this period and as a consequence were not captured, and it is further not unlikely that such early recurrent events could have influenced the decision of when to initiate DOAC treatment, therefore in 2 ways acting as a hidden confounder in the observational cohort. This may also explain the difference in Kaplan–Meier slope appearance between the observational and randomised populations seen in the earliest period after the event (Figures 1 and 2).

Death during hospitalisation was common among the patients with atrial fibrillation and ischaemic stroke registered in Riksstroke. Patients who died during the index hospitalisation, and patients who lacked dates for stroke onset or DOAC initiation were not included in the analysis, as neither of these had sufficient data for group assignment (Figure S1). This may have introduced bias in the analysis, as these circumstances could be more common in patients with negative outcomes, and death being a negative outcome in itself. The lack of randomisation in this population, where decisions on DOAC initiation were at the discretion of the treating physician, may additionally have introduced unmeasured bias in the analysis. Caution must thus be taken when interpreting the results from the observational cohort.

## Conclusion

This 1-year follow-up of the randomised TIMING study confirms the safety and efficacy of early initiation of DOAC therapy in patients with acute ischaemic stroke and atrial fibrillation. The similarity in patient characteristics and outcome event rates between the randomised study and the observational cohort emphasise the applicability of the results for routine practice.

## Supplementary Material

aakag010_TIMING_1year_outcomes_suppl_FINAL_ESJ

## Data Availability

Access to the data used in this study is restricted by the terms of the ethical review decisions and by applicable Swedish laws. On reasonable request, the authors may provide additional summary data, as well as metadata and technical documents underlying the statistical analyses.

## Trial registration

Unique identifier: NCT02961348 (http://www.clinicaltrials.gov).
