# Supplementary material for: Early versus delayed DOAC after ischaemic stroke in atrial fibrillation: 1-year outcomes in the TIMING study and in concurrent practice
Source: Eur Stroke J. 2026 Feb 27;11(2):aakag010. doi: 10.1093/esj/aakag010 (PMC12947707; doi:10.1093/esj/aakag010)

Early versus delayed DOAC after ischaemic stroke in atrial fibrillation: 1-year outcomes in the TIMING study and in concurrent practice

Supplemental material

# Table of contents

Tables 3

Table S1. 90 day outcomes in the observational cohort 3

Figures 4

Figure S1. STROBE diagram, observational cohort 4

Figure S2. Kaplan-Meier plot of all-cause mortality, TIMING population 4

Figure S3. Kaplan-Meier plot of all-cause mortality, observational cohort 5

Figure S4. Kaplan-Meier plot of recurrent ischemic stroke, TIMING population 6

Figure S5. Kaplan-Meier plot of recurrent ischemic stroke, observational cohort 7

Figure S6. Kaplan-Meier plot of symptomatic intracranial hemorrhage, observational cohort 8

# Tables

## Table S1. Outcomes at 90 days, observational cohort

|  | Observational cohort | | | TIMING study |
| --- | --- | --- | --- | --- |
|  | All (n=8951) | Early (n=6671) | Delayed (n=2280) | All (n=888) |
| Primary composite outcome within 90 days | 711 (7.9%) | 486 (7.3%) | 225 (9.9%) | 69 (7.8%) |
| Recurrent ischemic stroke within 90 days | 143 (1.6%) | 115 (1.7%) | 28 (1.2%) | 34 (3.8%) |
| Symptomatic intracerebral hemorrhage within 90 days | 10 (0.1%) | 8 (0.1%) | 2 (0.09%) | 0 (0.0%) |
| All-cause mortality within 90 days | 596 (6.7%) | 393 (5.9%) | 203 (8.9%) | 46 (5.2%) |
| Any major haemorrhages within 90 days | 234 (2.6%) | 176 (2.6%) | 58 (2.5%) | 30 (3.4%) |
| Re-admission to hospital within 90 days | 1961 (21.9%) | 1457 (21.8%) | 504 (22.1%) | 169 (19%) |

# Figures

## Figure S1. STROBE diagram, observational cohort


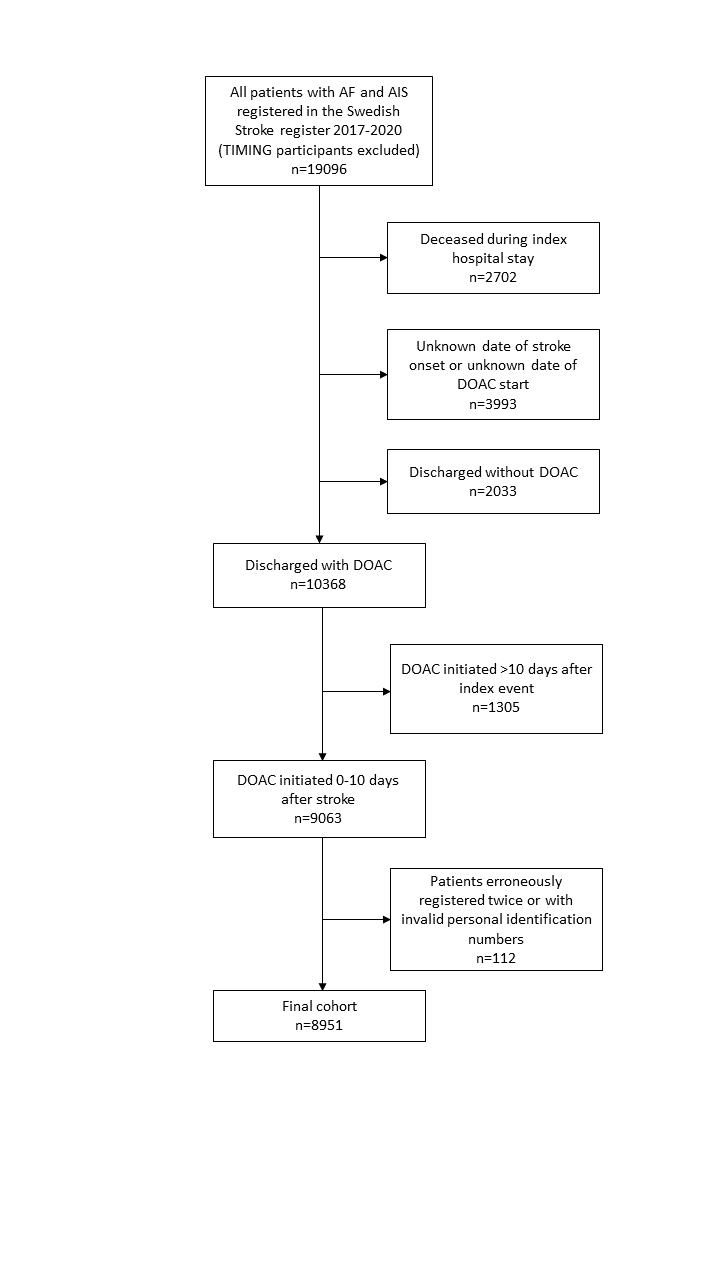


Figure S2. Kaplan-Meier plot of all-cause mortality, TIMING population


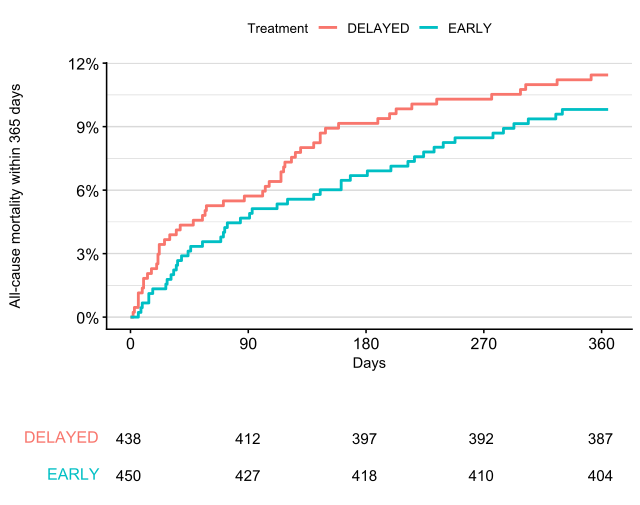


## Figure S3. Kaplan-Meier plot of all-cause mortality, observational cohort


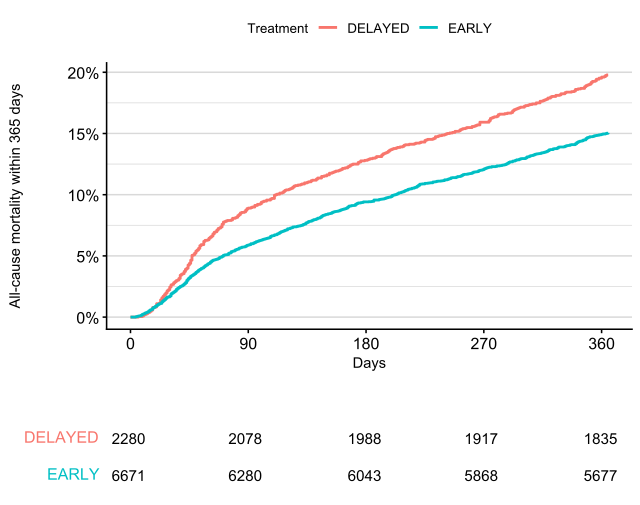


## Figure S4. Kaplan-Meier plot of recurrent ischemic stroke, TIMING population


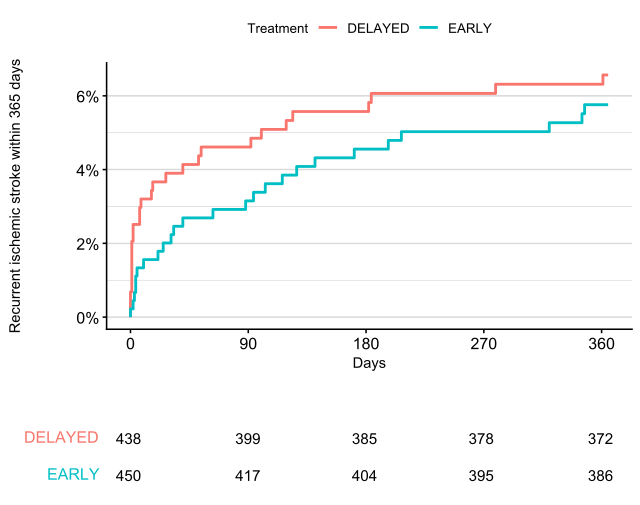


## Figure S5. Kaplan-Meier plot of recurrent ischemic stroke, observational cohort


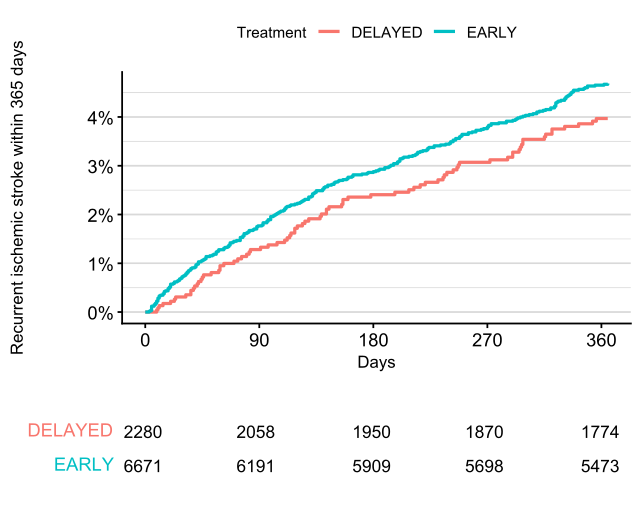


## Figure S6. Kaplan-Meier plot of symptomatic intracranial hemorrhage, observational cohort


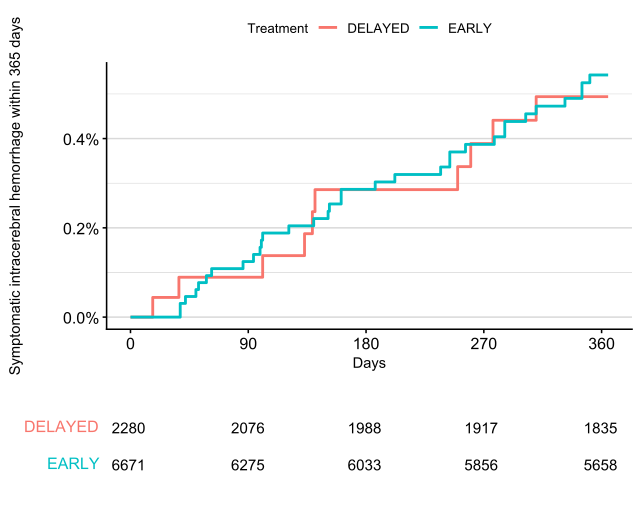

Supplement: aakag010_TIMING_1year_outcomes_suppl_FINAL_ESJ [file aakag010_timing_1year_outcomes_suppl_final_esj.docx]
